# Effects of low-frequency rTMS combined with speech and language therapy on Broca’s aphasia in subacute stroke patients

**DOI:** 10.3389/fneur.2024.1473254

**Published:** 2024-10-30

**Authors:** Li Gan, Litao Huang, Yin Zhang, Xin Yang, Lijuan Li, Lijiao Meng, Quan Wei

**Affiliations:** ^1^Department of Adult Speech and Swallowing Therapy, Affiliated Sichuan Provincial Rehabilitation Hospital of Chengdu University of TCM, Chengdu, China; ^2^Rehabilitation Medicine Center and Institute of Rehabilitation Medicine, West China Hospital, Sichuan University, Chengdu, China; ^3^Key Laboratory of Rehabilitation Medicine in Sichuan Province, Sichuan University, Chengdu, China; ^4^Department of Clinical Research Management, West China Hospital of Sichuan University, Chengdu, China; ^5^Health and Rehabilitation College, Chengdu University of TCM, Chengdu, China

**Keywords:** stroke, Broca’s aphasia, functional near-infrared spectroscopy, repetitive transcranial magnetic stimulation, speech and language therapy

## Abstract

**Introduction:**

Broca’s aphasia is a crushing syndrome after stroke. Although there are multiple therapies, the recovery of a considerable number of patients is still not ideal. Repetitive transcranial magnetic stimulation (rTMS) combined with speech and language therapy has been a promising combination regimen in recent years. However, the efficacy and persistent effects thereof remain unclear. We aimed to determine the immediate and long-term effects of rTMS combined with speech and language therapy on subacute stroke patients with Broca’s aphasia and explore relevant mechanisms in the picture-naming task via functional near-infrared spectroscopy (fNIRS).

**Materials and methods:**

This was a prospective clinical study. In accordance with the inclusion criteria, 18 patients with post-stroke were recruited and randomly divided into either the rTMS group or the sham-rTMS group. Patients in both groups received low-frequency rTMS therapy for 20 min a day and then speech and language therapy for 30 min a day, 5 days a week, for a total of 4 weeks. Two groups of patients underwent the Western Aphasia Battery Revised (WAB-R), the Stroke and Aphasia Quality of Life Scale-39 (SAQOL-39), and non-language-based cognitive assessment (NLCA) before treatment and at 2 weeks, 4 weeks, and 3 months after treatment. Meanwhile, we collected fNIRS task state data while naming images before and after 4 weeks of treatment. The primary outcome was WAB-R changes. The secondary outcomes include the SAQOL-39, NLCA, as well as the difference in activation status of brain regions in the cortical language function network.

**Results:**

For the index scores of the two groups, the results of repeated-measures ANOVA indicated an increasing trend at three time points, i.e., after 2 weeks of treatment, 4 weeks after treatment, and 3 months after the end of treatment (*p* < 0.001); in terms of intergroup effects, there was a statistically significant difference between the two groups in WAB naming scores (*F* = 4.865, *p* = 0.042); and the aphasia quotient (AQ), listening comprehension, and naming scores of the two groups had interactive effects (*F*AQ = 11.316, *P*AQ = 0.000; *F*_listening_ = 8.205, *P*_listening_ = 0.002; *F*_naming_ = 27.46, *P*_naming_ = 0.000). Independent sample t-tests also showed that until 4 weeks after the end of treatment, there were significant differences in information volume and naming scores between the two groups (*t*_information_ = 2.352, *P*_information_ = 0.032; *t*_naming_ = 3.164, *P*_naming_ = 0.006). Three months after the end of treatment, there were significant differences in information volume, naming, AQ and repetition scores (*t*_information_ = 2.824, *P*_information_ = 0.012; *t*_naming_ = 5.090, *P*_naming_ = 0.000; *t*AQ = 2.924, *P*AQ = 0.010; *t*_repetition_ = 2.721, *P*_repetition_ = 0.015). In the picture-naming task, fNIRS analysis found that in the rTMS group after treatment, the activation in the left superior temporal gyrus (STG), middle temporal gyrus (MTG), premotor cortex (PM), supplementary motor area (SMA), pars triangularis Broca’s area, and dorsolateral prefrontal lobe (DLPFC) decreased (*p* < 0.05).

**Conclusion:**

The language function of patients was improved after 4 weeks of treatment, and there was a long-term effect (3 months follow-up), especially in naming gains. Moreover, by analyzing cortical activation during a picture-naming task with fNIRS, we found that rTMS could downgrade the activation level in the left MTG, STG, PM and SMA, DLPFC, and pars triangularis Broca’s area, whereas the sham-rTMs group only showed downgraded activation levels in the right PM and SMA. This demonstrates the unique mechanism of rTMS.

**Clinical trial registration**: ChiCTR.org.cn, identifier, ChiCTR2300067703.

## 1 Introduction

Post-stroke aphasia is one of the most serious complications among stroke survivors (1, 2), with clinical manifestations including loss or impairment of one or more aspects of listening comprehension, oral expression, retelling, naming, reading, and writing, to varying degrees (3). The incidence of aphasia after the first stroke is 32% (4), up to 40% of speech disorders persist after 1 year (5), and residual speech symptoms may affect patients’ lives for many years.

Approximately 20% of post-stroke aphasia patients cannot reach the most basic level of daily communication after speech therapy (6). Broca’s aphasia, the classical subtype of non-fluent aphasia, is characterized by oral expression disorders and caused by lesions involving the Broca areas in the left inferior frontal gyrus (dominant hemisphere). Speech and language therapy is the most recommended rehabilitation approach for aphasia (1), which reduces language deficits, but only results in limited curative effects and needs long rehabilitation periods (7). To date, effects of pharmacological therapies for Broca’s aphasia are also limited (8, 9). The prognosis for post-stroke aphasia is, therefore, currently not ideal, and the efficacy of speech and cognitive therapy alone is inadequate.

When repetitive transcranial magnetic stimulation (rTMS) is used alone, it can produce substantial improvements in language and cognitive abilities in post-stroke aphasia patients in the chronic or subacute stage (10–12). Furthermore, rTMS combined with speech and language therapy may provide added benefits over speech and language therapy alone in these stages (13–15). There is also a lack of research on rTMS in Broca’s aphasia or other subtypes thereof (16), especially in the subacute phase of stroke, which makes it difficult to provide conclusive recommendations on whether integrating rTMS with speech and language therapy is advisable for stroke patients with Broca’s aphasia in the subacute phase.

In recent years, functional near-infrared spectroscopy (fNIRS) has been recognized as a promising imaging technology for the study of brain function (17, 18). Through real-time detection of the oxyhemoglobin and deoxyhemoglobin concentrations in the cerebral cortex, neural activity in the brain can be estimated using fNIRS (19, 20). Additionally, the effects of naming tasks observed with fNIRS in healthy individuals and nonaphasics have been reported (19, 21–23), but the research only involved brain regions such as the STG, MTG, premotor cortex, and SMA, not including other important language and cognitive brain regions (e.g., Wernicke area, pars triangularis Broca’s area, and the dorsolateral prefrontal cortex).

We, therefore, aimed to target subacute-stage stroke patients with Broca’s aphasia, and apply low-frequency rTMS combined with speech and language therapy to determine immediate and long-lasting effects of these treatments. We also explored the changes in the activation degree of specific language and cognitive brain regions during a picture-naming task before and after treatment with fNIRS.

## 2 Methods

### 2.1 Design

**Figure 1:**
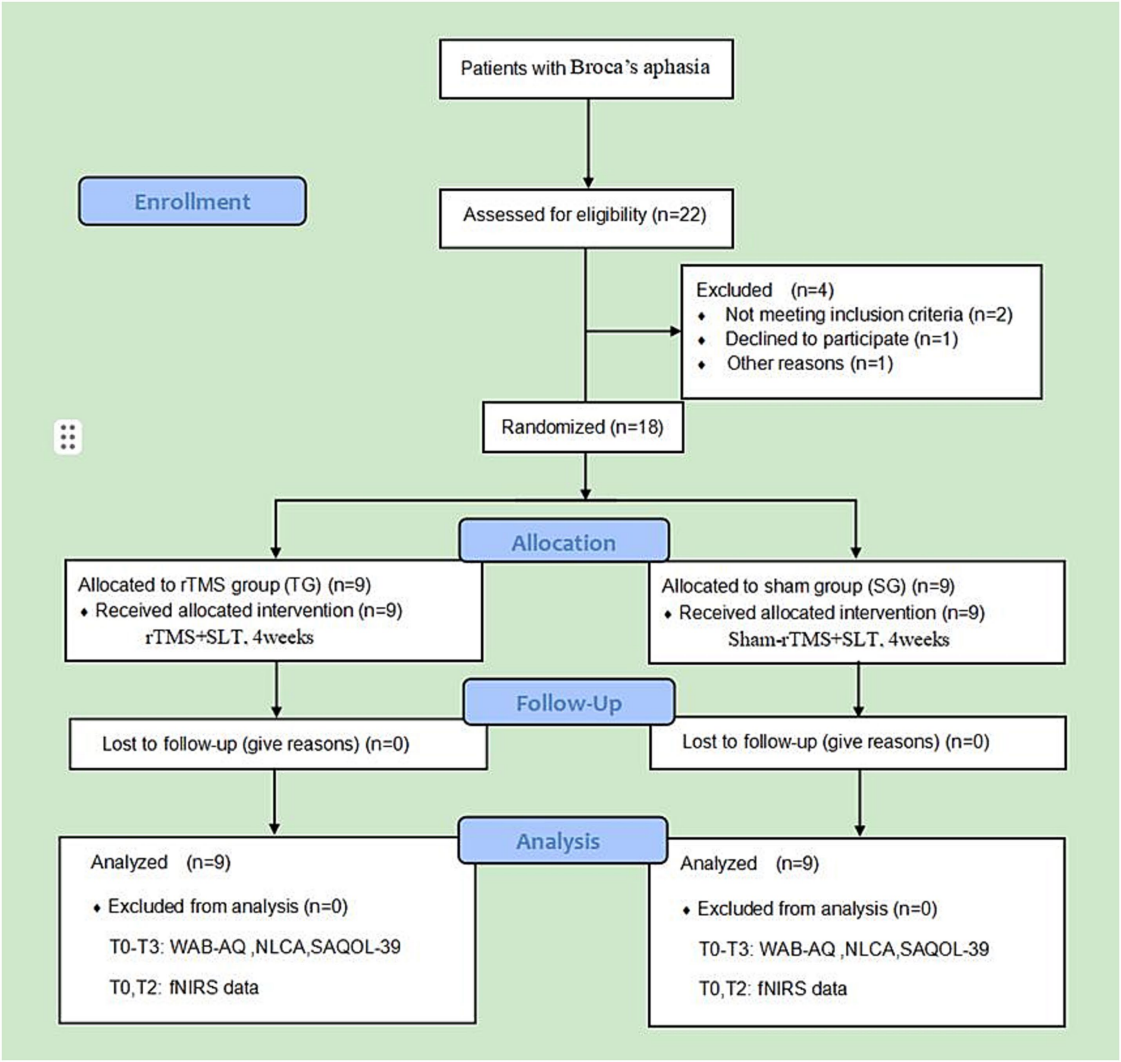
Consolidated Standards of Reporting Trials (CONSORT) subject flow diagram. T0, before treatment; T1, after 2 weeks of treatment; T2, after 4 weeks of treatment; T3, at 3-month follow-up.

Our study was a randomized, single-center, double-blinded, sham-controlled trial. The study protocol followed the Consolidated Standards of Reporting Trials (CONSORT) guidelines, as well as the Recommendations for Interventional Trials (SPIRIT). The study was conducted at the Sichuan Rehabilitation Hospital (Sichuan Bayi Rehabilitation Center) in Chengdu, China. All procedures were reviewed and approved by the Medical Ethics Committee of Sichuan Bayi Rehabilitation Center (CKLL-2022014). This study was registered at the Chinese Clinical Trial Registration Center (No. ChiCTR2300067703). The research protocol and related documents can be obtained through ClinicalTrials.gov and the corresponding authors. The intervention cycle was 4 weeks, and the follow-up period was 3 months after the end of treatment. The clinical language and cognitive function indices, changes in cerebral cortex activation during the task, and therapeutic effects during the follow-up period were compared and analyzed. All patients completed corresponding assessments before and after treatment, as well as during the 3-month follow-up. Figure 1 shows the specific study details.

### 2.2 Participants

#### 2.2.1 Inclusion, exclusion, and withdrawal criteria

We included first-onset supratentorial stroke patients who met the diagnostic criteria of the Chinese Guidelines for Clinical Management of Cerebrovascular Diseases (2nd edition) (24); diagnosed with Broca’s aphasia revealed using the Chinese version of the Western Aphasia Rating Scale; conscious and stable patients with and had no serious cognitive dysfunction, older than 18 years; course of disease 1–6 months; years of education ≥6 years; right-handed, assessed via the Edinburgh Handedness Scale (score > +40); native speakers of Chinese; no history of language disorders before onset; complete clinical language cognitive function examination, fNIRS examination, speech therapy, and rTMS treatment; and patients or family members signed informed consent forms.

We excluded patients with speech disorders caused by peripheral sensory (e.g., visual, auditory, etc.) abnormalities (25); complicated by heart, liver, or kidney dysfunction and other serious diseases; patients with complications such as epilepsy or other neurological diseases, such as motor neuron disease and Parkinson’s disease; those with a history of neurological or organic mental illness; intracranial metal internal fixation and other devices; and patients with fNIRS signal acquisition affected by skull defects in the brain, hair occlusion, and other factors (22, 26).

Participants could quit the study voluntarily or were withdrawn when serious adverse events, such as seizures and stroke recurrence, occurred.

#### 2.2.2 Sample size

This study is an exploratory prospective clinical study. Previous studies usually included 5–10 participants (19, 21) with aphasia. Referring to the latest study design (19, 21, 23, 25, 27), we used G*Power software to calculate the sample size with a type I error of 0.05 (*α*) and a type II error of 0.80 (1-*β*). Additionally, a 20% dropout rate was considered. A total of 22 participants were recruited, and four patients were excluded according to the inclusion and exclusion criteria.

### 2.3 Randomization and blinding

The random serial numbers of 18 patients were generated by SPSS software and placed into sequentially coded sealed and opaque envelopes. After the researchers had determined the eligibility of the participants, the envelopes were opened in order, and the participants were randomly assigned to one of the two groups (including nine patients in the rTMS group and nine patients in the sham group). The participants, speech therapists, evaluators, and data statisticians involved in this study were blinded.

### 2.4 Assessments

#### 2.4.1 Language function assessment and cognitive function screening

A speech therapist with more than 5 years of work experience, who was not aware of the groups, assessed the severity of language ability and the quality of life of post-stroke aphasia patients before treatment, 2 weeks after treatment, 4 weeks after treatment, and at the 3-month follow-up. The specific assessment includes the following aspects:

1. Western Aphasia Battery Revised (WAB-R). The WAB scale was used to record the overall language ability of patients with post-stroke aphasia before and after treatment, namely, the aphasia quotient (AQ), with a total score of 100. Subscores for naming, repetition, listening comprehension, fluency, information content, and other subitems were collected. The total score of each subitem was 10 (21).
2. The Stroke and Aphasia Quality of Life Scale-39 (SAQOL-39) was used to evaluate the activity and participation ability of post-stroke aphasia patients. The content covers three aspects of physiology, psycho-society, and communication, with a total of 39 subitems, and each item is graded at five levels (28).
3. Non-language-based cognitive assessment (NLCA). The NLCA is widely used to assess the overall non-verbal cognitive function of aphasia patients and has high sensitivity and specificity to identify aphasia patients with mild cognitive impairment. It mainly involves visuospatial ability, memory, attention, logical reasoning ability, and executive ability. The maximum possible score is 80 points. A score lower than 70 points indicates cognitive dysfunction (29, 30).

#### 2.4.2 fNIRS data collection preparation

**Figure 2:**
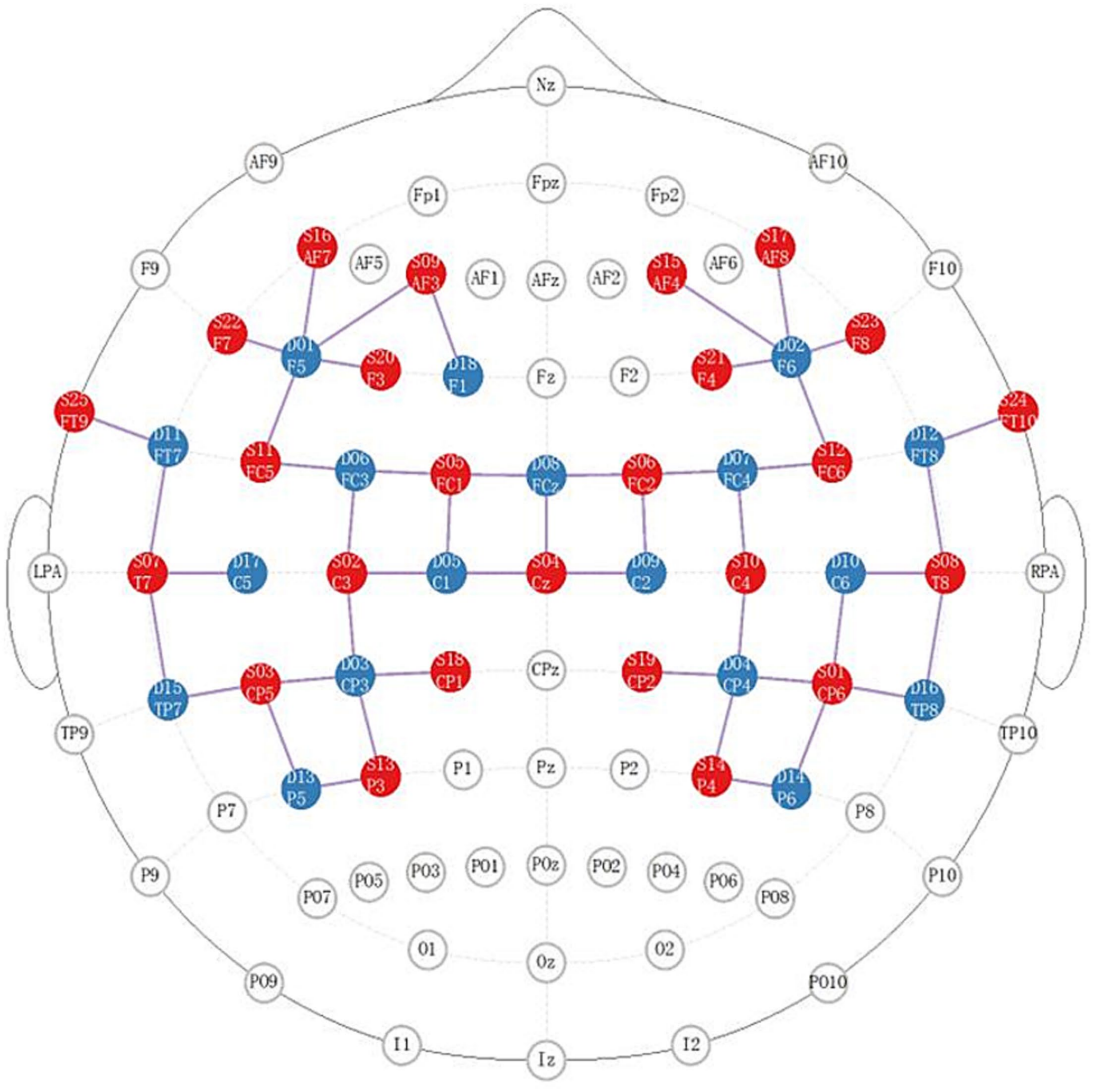
Distribution diagram of the light source and detector in the brain area. The red area indicates the light source; the blue area indicates the detector; the lines indicate the channels.

**Figure 3:**
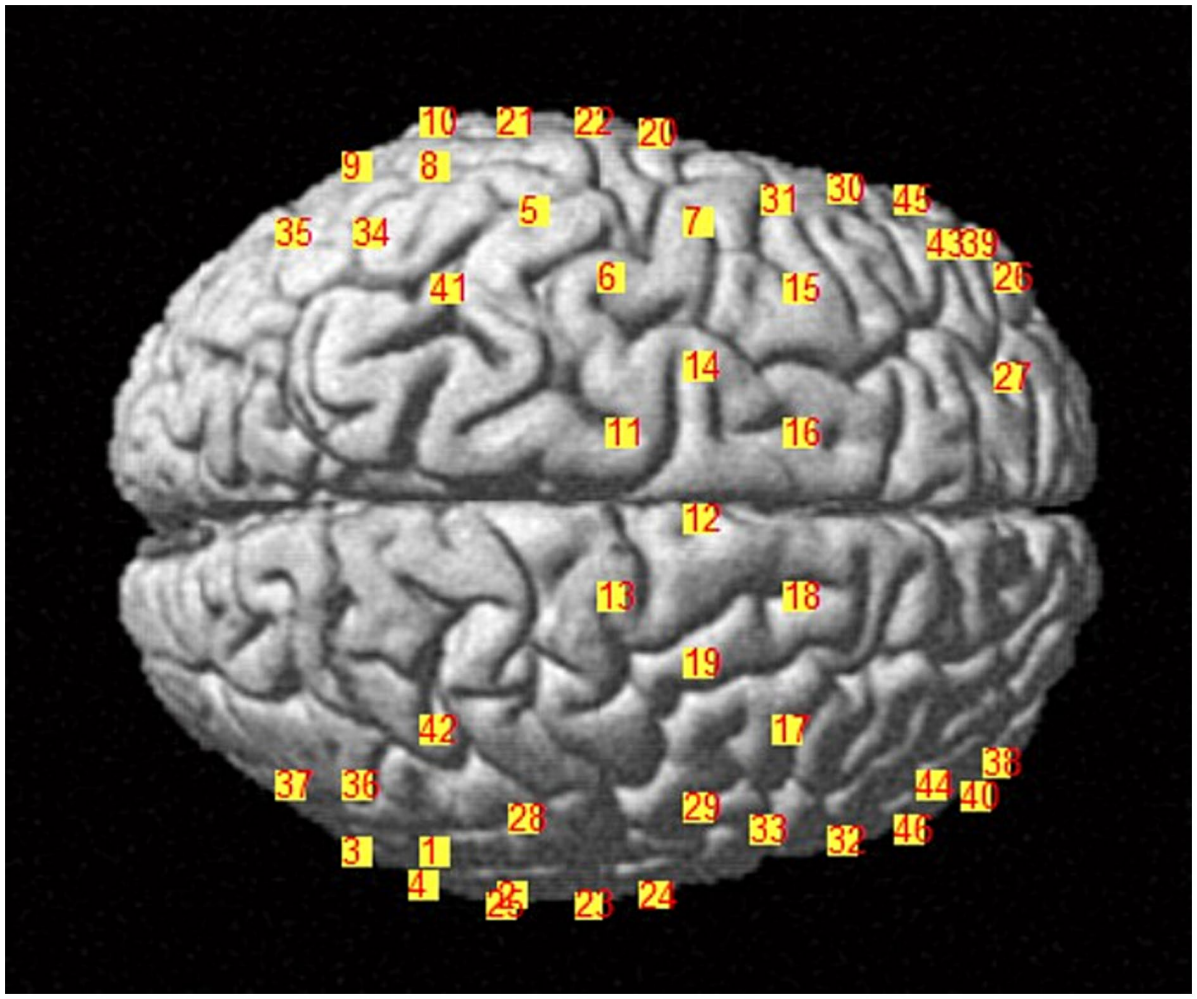
Distribution diagram of 48 channels in the brain.

We applied NIRX fNIRS imaging equipment (NIRx Medical Technologies, NY, USA) with a sampling frequency of 2.5 Hz and a continuous wave recording of two different wavelengths of near-infrared light (785, 830 nm) signals (31). According to our design, a total of 48 channel signals from the left and right brain hemispheres were collected from each patient, the optical pole included 25 light source transmitters and 18 detectors, and the average distance between the detectors and the light source was 30 mm (31, 32). The main detection area of each channel is located in the brain region below the midpoint of the channel (21, 33, 34), the coordinate information of the middle point of the channel is determined by a Patriot Locator (21), and the collection head cap is designed based on the international 10–20 standard electrode placement system (32, 35) (Figure 2). The NIRS-SPM toolkit was used to convert the spatial coordinate system of the midpoint of each pair of light sources and detectors into 48-channel MNI coordinates of the Montreal Neurological Institute, and the corresponding information between the MNI coordinates and Brodmann partition location was obtained (31, 32). The BrainNetViewer toolbox was subsequently used for visualization (21, 36, 37) (Figure 3). The 48 channels were divided into six ROIs (21) in the cerebral cortex of the participants, and the left and right sides were distinguished. The channel information corresponding to each ROI is shown in Table 1.

**Table 1:** Channel distribution of the ROI.

ROI	Left cerebral channel	Right cerebral channel
Broca area	30	32
Wernicke area	5, 8, 9, 34, 35	1, 2, 3, 28, 36, 37
DLPFC	27, 31, 43	33, 44
STG	10, 22	4, 23
MTG	20, 21, 48	24, 25, 47
SMA	6, 7, 11, 14, 15, 16	12, 13, 17, 18, 19, 29

ROI, region of interest; DLPFC, dorsolateral prefrontal cortex; STG, superior temporal gyrus; MTG, middle temporal gyrus; SMA, supplementary motor area.

### 2.5 fNIRS task state data collection

Both groups received language training for 4 weeks, 30 min a day, 5 days a week, for 4 weeks. Data collection during the fNIRS picture-naming task was performed within 48 h after the two groups were enrolled and within 48 h after the end of 4 weeks of treatment, mainly to observe and record the changes in cerebral cortex activation in patients in the task state.

**Figure 4:**
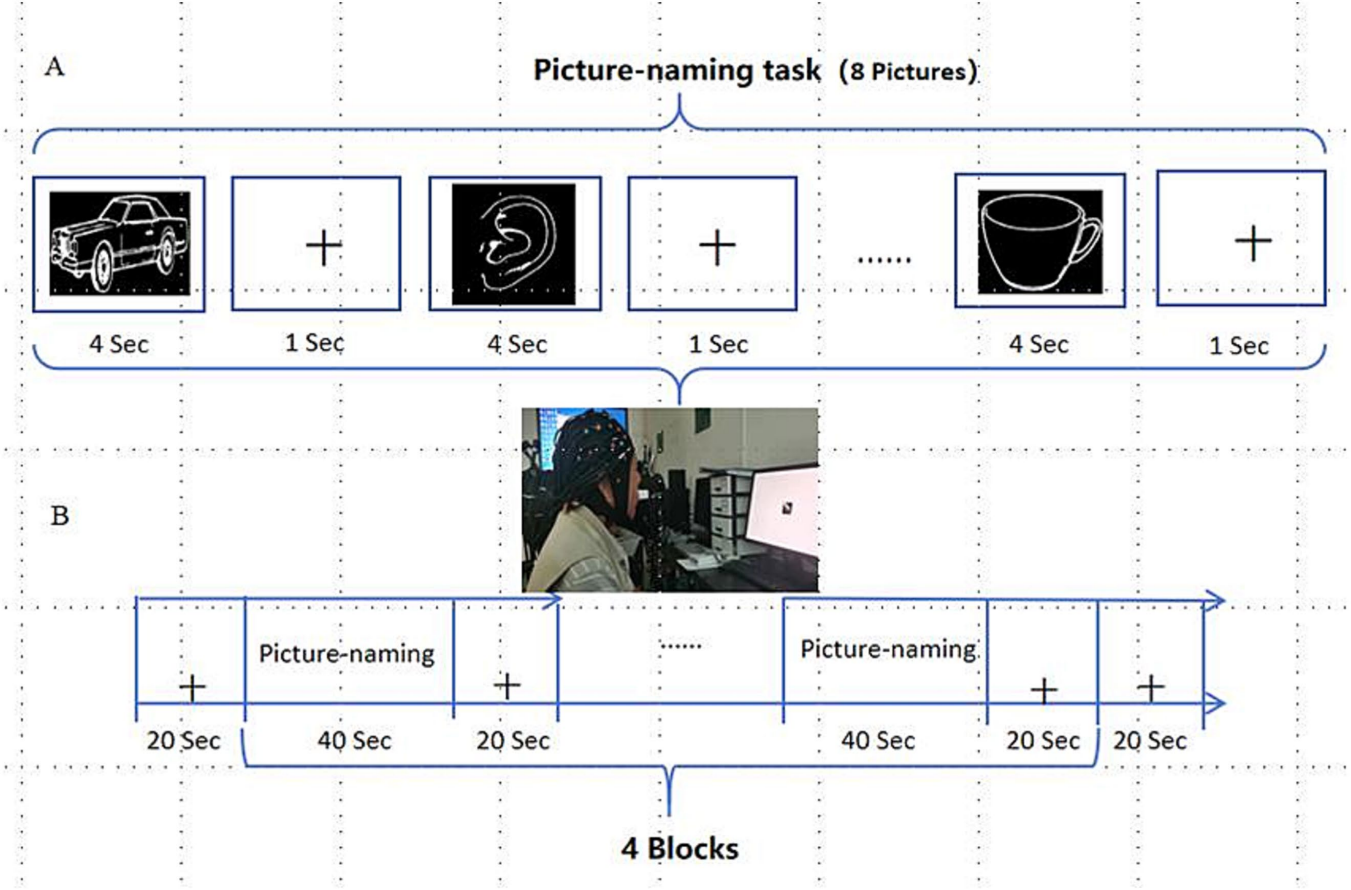
Picture-naming task. (A) Picture-naming task paradigm; (B) Periodic experimental design for picture naming.

E-prime software was used to program and carry out image naming experimental tasks, and the patients completed corresponding tasks according to the instructions on the screen. According to the setting of word frequency and difficulty level, 32 black and white pictures from the psychological cognitive experiment picture database (21) were selected. The task pattern was designed with periodic blocks, including 40 s experimental blocks and 20 s control blocks. Eight pictures of the standby names were presented in each periodic block task, and each picture was presented for 4 s. Participants then entered the rest of the period and stared at the black “cross” on the computer screen for 1 s until the next picture appeared (Figure 4).

### 2.6 Interventions

#### 2.6.1 Speech and language therapy protocol

Based on the Schuell therapy technique, the principles of Schuell guided us to utilize the strong auditory stimulus, appropriate, multichannel language stimulation, and emphasized the importance of reinforcing correct response in time. The speech and language therapy protocol sessions were led by speech therapists and were tailored to subacute aphasia patient needs and abilities (38), including listening comprehension training, auditory memory span training, naming training, oral expression training, practical communication training, etc. (39). After rTMS treatment, patients rested for 5–10 min and received subsequent speech and language therapy. The treatment was formulated with reference to the consensus of rehabilitation experts for Chinese aphasia: 30 min a day, 5 days a week, for a total of 20 times, for 4 weeks (40–42). Moreover, the practicable design of this program is in line with current one-to-one treatment in most hospitals and rehabilitation institutions in China.

#### 2.6.2 rTMS protocol

A transcranial magnetic stimulation instrument (CCY-type, Classic Magnetic Stimulator, YIRUIDE GROUP, China) was used to carry out the therapeutic intervention in this study as follows. (1) Parameter selection: inhibitory stimulation with a low frequency of 1 Hz and an intensity of 90% of the resting motor threshold was selected (16, 43); stimulation time = 10 s, interstimulus interval = 2 s, repetition times = 100 times, 20 min a day, with a total of 1,000 pulses (44); (2) coil selection: the figure-of-eight coil; (3) stimulation site: right inferior frontal gyrus triangle (43) (i.e., pars triangularis, part of the Brodmann area 45), where the center of the figure-of-eight coil is placed at the mark, and the center is adjusted to be tangent to the patient’s skull, orienting the coil 90° toward the patient’s occiput, with the coil position fixed during each treatment session (27, 45). The standard electrode placement method 10–20 system (46), recognized by the International Electroencephalography Society, was used to determine the stimulation site, which is located at the intersection of the F8 and Cz lines and the T4 and FZ lines in the right cerebral hemisphere; (4) Resting motor threshold measurement: The patient was asked to relax, sit quietly, and place the recording electrodes on the abductor pollicis brevis muscle on the left upper limb. The reference electrode was placed on the first metacarpophalangeal joint. The coil is placed on the M1 region of the right cerebral hemisphere. After slightly adjusting the coil position and further determining the stimulation site that can induce the largest amplitude and the shortest latency, the output intensity was gradually reduced, and the minimum stimulus intensity that can trigger the contralateral abductor pollicis brevis motor evoked potential ≥50 μV for ≥5 consecutive stimuli was the motor threshold (47). (5) Treatment prescription: 20 min/day, 5 days a week, for a total of 20 sessions (16, 44), based on the results of previous pilot experiments, determined the 4-week stimulation period.

#### 2.6.3 Sham-rTMS protocol

When the coil is placed at the stimulation site, the stimulation surface is placed 90° perpendicular to the target area (48), and the rest of the stimulation surface is the same as that used in the rTMS treatment regimen described above.

The intervention period was 4 weeks (during hospitalization) for both real and sham-rTMS treatments. After discharge from the hospital, the patients did not receive any speech therapy and rTMS treatments. We assessed the patient’s language function every 2 weeks via phone or video conference and then gave guidance or instructions to family members.

### 2.7 Data preprocessing

#### 2.7.1 Language and cognitive function assessment data

The WAB AQ score (the main outcome indicator) and WAB subitems, including fluency, information content, listening comprehension, repetition, and naming, were selected as the observation indicators of language function in this study. The NLCA and SWQOL-39 were selected as the cognitive function and language-related functional indicators, respectively. The original scores of all data were recorded as per the evaluation checklist, the original paper data were registered in the evaluation manual and case report form, and the electronic versions were sorted and saved.

#### 2.7.2 fNIRS task state data

The NIRS-SPM toolkit (35, 49), running on the MATLAB platform, was used for data preprocessing, model construction, and analysis of the fNIRS data in the task state. The detailed steps are as follows: (1) the wavelet-MDL method was used for nonlinear trend and high-pass filtering; (2) a smoothing method with a hemodynamic response function (HRF) was used for low-pass filtering; (3) the precoloring method was used to estimate and remove the time domain correlation; (4) after preprocessing, the generalized linear model was constructed to estimate the HRF. The basis function hrf (time & dispersion der.) was selected. The presentation time of the first image of the first block of the named task was set to time 0 (onset time); (5) we selected 12 ROIs (six in each hemisphere), i.e., Broca area, Wernicke area, MTG, STG, SMA, dorsolateral prefrontal cortex. The regression coefficient beta values under each channel and ROI were obtained and used in the next statistical analysis beta values mainly reflect the analysis of the time domain, including the estimation of the amplitude change and the time to reach the peak. By calculating the changes in the amount of attenuation, the changes in oxygenated hemoglobin concentration in different regions of the brain can be indirectly measured. (6) The channel coverage indicates the percentage of cortical anatomical positions overlapped by different channels in the Brodmann area with reference to the Talairach Daemon Database.

### 2.8 Statistical analysis

#### 2.8.1 General information and language cognitive function indices

SPSS 25.0 statistical software was used to compare age, sex, disease course, education level, WAB AQ score, and other general data between the two groups. Independent two-sample t tests were used, and chi-square tests were used for sex comparisons and stroke site distribution comparisons. The WAB (AQ and subitems) scores, NLCA scores, and SAQOL-39 scores before treatment, 2 weeks, 4 weeks after treatment, and at 3 months were analyzed by repeated-measures ANOVA. At each time point, an independent sample t test (two tailed) was used to compare the two groups. *p* < 0.05 indicated a statistically significant difference.

#### 2.8.2 fNIRS task state data

The fNIRS data of the two groups were collected by ttest and ttest2 functions in MATLAB before and 4 weeks after treatment, and the beta values of each channel calculated by a model were tested by an in-group paired t test and an intergroup independent sample t test, respectively. For a two tailed test, *p* < 0.05 indicated a statistically significant difference.

## 3 Results

### 3.1 General condition

A total of 18 patients with Broca’s aphasia who met the inclusion criteria were recruited from the Neurorehabilitation Department of Sichuan Rehabilitation Hospital between January 2023 and December 2023. The participants were randomly assigned to the rTMS group (*n* = 9) or the sham group (*n* = 9) at a 1:1 ratio. During the study period, all patients completed the assessment, treatment, and follow-up, and no adverse events occurred. All clinical data and evaluation data of the two groups were complete. The differences in general information and language cognitive function indices were not statistically significant (*p* > 0.05) (Tables 2, 3).

**Table 2:** Demographic data between the rTMS group and the sham group.

Group	Cases	Sex		Age (years)	Course of disease (days)	Education (years)	Apoplexy site (case)			
		Male	Female				Temporal lobe	Frontal lobe	Basal ganglia	Multiple
TG	9	9	0	48.11 ± 11.08	91.67 ± 47.91	12.00 ± 3.12	3	1	2	3
SG	9	8	1	48.78 ± 12.67	92.44 ± 56.89	12.56 ± 3.28	2	2	2	3
*t/x^2^* value		0.000		−0.119	−0.031	−0.368	0.912			
*p* value		1.000^a^		0.907^b^	0.975^b^	0.718^b^	0.533^a^			

a Chi-square test.

b Independent two-sample t test.

TG, rTMS group; SG, sham group.

**Table 3:** Language and cognitive functioning between the rTMS group and the sham group (scores).

Group	Cases	AQ	SAQOL-39	NLCA	Fluency	Inform.	Listen.	Repetition	Naming
TG	9	39.23 ± 22.78	94.33 ± 33.93	66.22 ± 13.14	2.67 ± 1.73	4.33 ± 2.78	5.43 ± 1.92	4.48 ± 3.17	2.71 ± 2.77
SG	9	39.22 ± 22.92	94.00 ± 26.17	65.11 ± 13.31	2.00 ± 1.73	3.89 ± 2.93	6.63 ± 2.30	4.44 ± 3.48	2.66 ± 3.12
*t/x^2^* value		0.001	0.023	0.178	0.816	0.330	−1.202	0.025	0.040
*p* value		0.999^b^	0.982^b^	0.861^b^	0.426^b^	0.746^b^	0.247^b^	0.981^b^	0.969^b^

b Independent two-sample t test.

TG, rTMS group; SG, sham group; inform., information volume; listen., listening comprehension.

### 3.2 Improvements in language and cognitive function

Before the intervention, there were no significant differences in the scores of language cognitive function between the two groups (*p* > 0.05). The results of the repeated-measures ANOVA showed that the scores of the above indices in the two groups tended to increase at three time points (after 2 weeks of treatment, 4 weeks after treatment, and 3 months after the end of treatment), and the differences were statistically significant (*p* < 0.001). In terms of intergroup effects, there was a statistically significant difference in naming scores between the two groups (*F* = 4.865, *p* = 0.042), but there was no statistically significant difference in scores among the other indicators (*p* > 0.05). The AQ, listening comprehension, and naming scores of the two groups had interactive effects (*F*_AQ_ = 11.316, *P*_AQ_ = 0.000; *F*_listening_ = 8.205, *P*_listening_ = 0.002; *F*_naming_ = 27.46, *P*_naming_ = 0.000).

**Figure 5:**
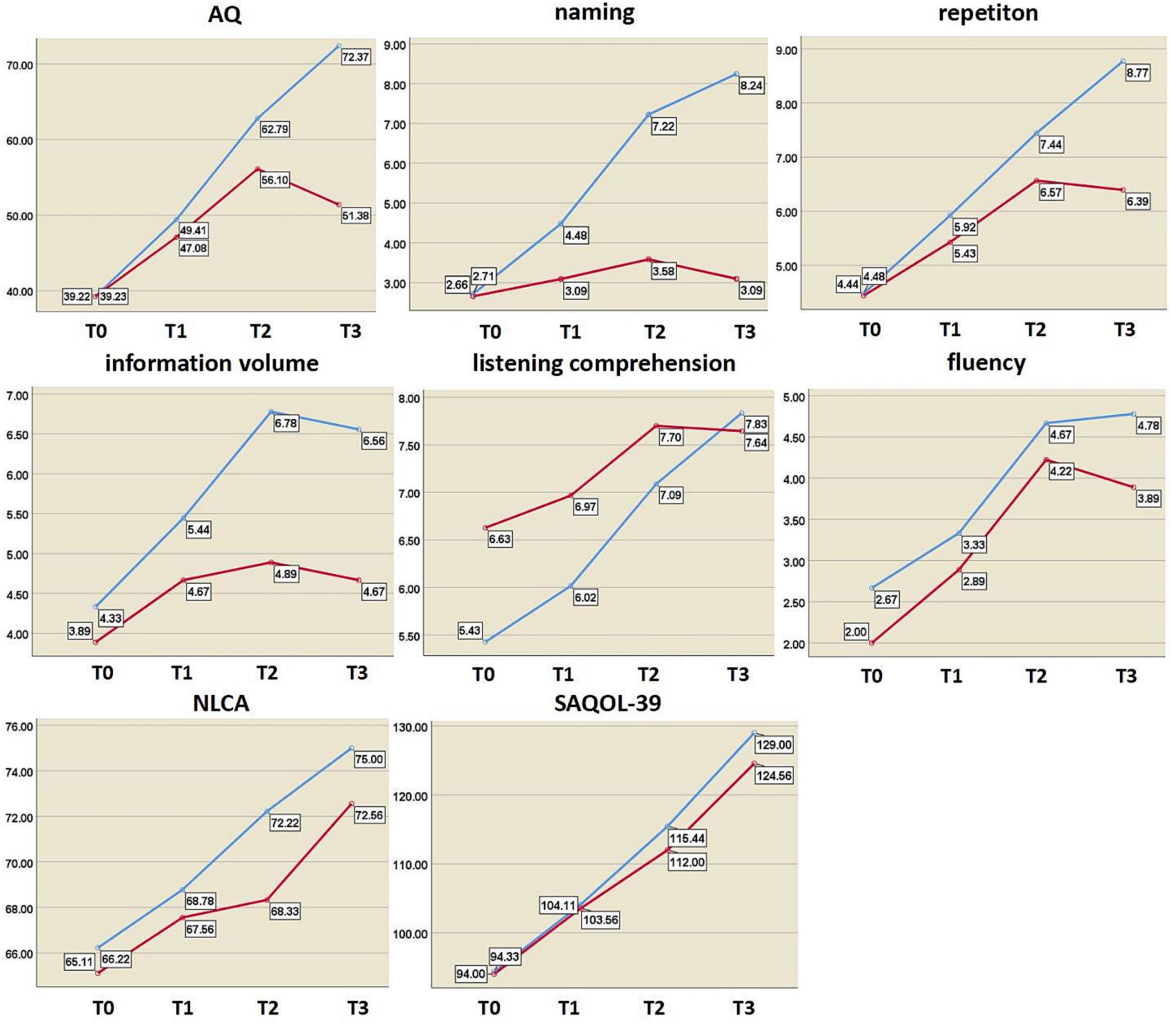
Changes in language and cognitive function indices at different time points. The *y* axis represents the score value of each index; blue curve, rTMS group; red curve, sham group; AQ, aphasia quotient; NLCA, non-language-based cognitive assessment; SAQOL-39, Stroke and Aphasia Quality of Life Scale-39; T0, before treatment; T1, after 2 weeks of treatment; T2, after 4 weeks of treatment; T3, at 3-month post-treatment.

Independent sample t-tests revealed no significant differences in any of the indicators between the two groups after 2 weeks of treatment (*p >* 0.05). After 4 weeks of treatment, there were statistically significant differences in information content and naming scores between the two groups (*t*_information_ = 2.352, *P*_information_ = 0.032; *t*_naming_ = 3.164, *P*_naming_ = 0.006). During the 3-month follow-up, there were significant differences in information volume, naming, AQ, and repetition scores (*t*_information_ = 2.824, *P*_information_ = 0.012; *t*_naming_ = 5.090, *P*_naming_ = 0.000; *t*_AQ_ = 2.924, *P*_AQ_ = 0.010; *t*_repeating_ = 2.721, *P*_repeating_ = 0.015) (Table 4 and Figure 5). Regardless of whether real rTMS was received or not, all eight indicators showed sustained improvement within 4 weeks. During the period of 4 weeks to 3 months, all six speech indicators except NLCA and SAQOL-39 in the sham-rTMS group deteriorated; on the contrary, in the rTMS group, except for information volume, the other five speech indicators not only showed continuous improvement within 4 weeks of treatment but also continued to improve during the period of 4 weeks to 3 months after stopping treatment. This finding suggests that the rTMS scheme used in this study not only has short-term effects but also unique long-term effects.

**Table 4:** Language and cognitive function indicators between the rTMS group and the sham group (*n* = 9, score).

Indicator	Group	Pre-treatment	Post-treatment			*F*_time_	*F*_interclass_	*F*_interaction_
			2 weeks	4 weeks	3 months	*P*_time_	*P*_interclass_	*P*_interaction_
AQ	TG	39.23 ± 22.78	49.41 ± 18.24	62.79 ± 14.96	72.37 ± 11.78			
	SG	39.22 ± 22.92	47.07 ± 21.41	56.10 ± 22.23	51.38 ± 18.03	48.011	0.706	11.316
	*t* value	0.001	0.249	0.749	2.924	0.000*	0.413	0.000*
	*p* value	0.999	0.807	0.465	0.010*			
NLCA	TG	66.22 ± 13.14	68.78 ± 10.17	72.22 ± 6.63	75.00 ± 4.56			
	SG	65.11 ± 13.31	67.56 ± 10.84	68.33 ± 11.69	72.56 ± 7.94	5.516	0.224	1.350
	*t* value	0.178	0.247	0.868	0.801	0.010*	0.642	0.298
	*p* value	0.861	0.808	0.392	0.435			
SWQOL-39	TG	94.33 ± 33.93	104.11 ± 26.57	115.44 ± 24.84	129.00 ± 21.62			
	SG	94.00 ± 26.17	103.56 ± 23.37	112.00 ± 24.03	124.56 ± 21.13	26.804	0.036	0.231
	*t* value	0.023	0.047	0.299	0.441	0.000*	0.853	0.874
	*p* value	0.982	0.963	0.769	0.665			
Fluency	TG	2.67 ± 1.73	3.33 ± 1.41	4.67 ± 1.80	4.78 ± 1.79			
	SG	2.00 ± 1.73	2.89 ± 2.03	4.22 ± 2.59	3.89 ± 2.03	12.747	0.517	0.713
	*t* value	0.816	0.539	0.423	0.987	0.000*	0.483	0.560
	*p* value	0.426	0.597	0.678	0.339			
Infor.	TG	4.33 ± 2.78	5.44 ± 2.19	6.78 ± 1.64	6.56 ± 1.51			
	SG	3.89 ± 2.93	4.67 ± 2.12	4.89 ± 1.76	4.67 ± 1.32	8.482	1.868	3.272
	*t* value	0.330	0.766	2.352	2.824	0.002*	0.191	0.053
	*p* value	0.746	0.455	0.032*	0.012*			
Listen.	TG	5.43 ± 1.92	6.02 ± 1.70	7.09 ± 1.29	5.43 ± 1.92			
	SG	6.63 ± 2.30	7.00 ± 2.10	7.70 ± 1.83	6.63 ± 2.30	17.791	0.623	8.205
	*t* value	−1.202	−1.057	−0.817	0.266	0.000*	0.441	0.002*
	*p* value	0.247	0.306	0.426	0.793			
Repetition	TG	4.48 ± 3.17	5.92 ± 2.66	7.44 ± 2.15	8.77 ± 1.04			
	SG	4.44 ± 3.48	5.43 ± 2.90	6.57 ± 3.01	6.39 ± 2.41	19.559	0.611	2.691
	*t* value	0.025	0.376	0.712	2.721	0.000*	0.446	0.086
	*p* value	0.981	0.712	0.487	0.015*			
Naming	TG	2.71 ± 2.77	4.48 ± 1.98	7.22 ± 1.56	8.24 ± 1.07			
	SG	2.66 ± 3.12	3.09 ± 2.98	3.58 ± 3.08	3.09 ± 2.84	30.783	4.865	27.46
	*t* value	0.040	1.165	3.164	5.090	0.000*	0.042*	0.000*
	*p* value	0.969	0.261	0.006*	0.000*			

*p < 0.05; TG, rTMS group; SG, sham group; Inform., information volume; Listen., listening comprehension.

### 3.3 Changes in cortical activation in the task state detected with fNIRS

**Figure 6:**
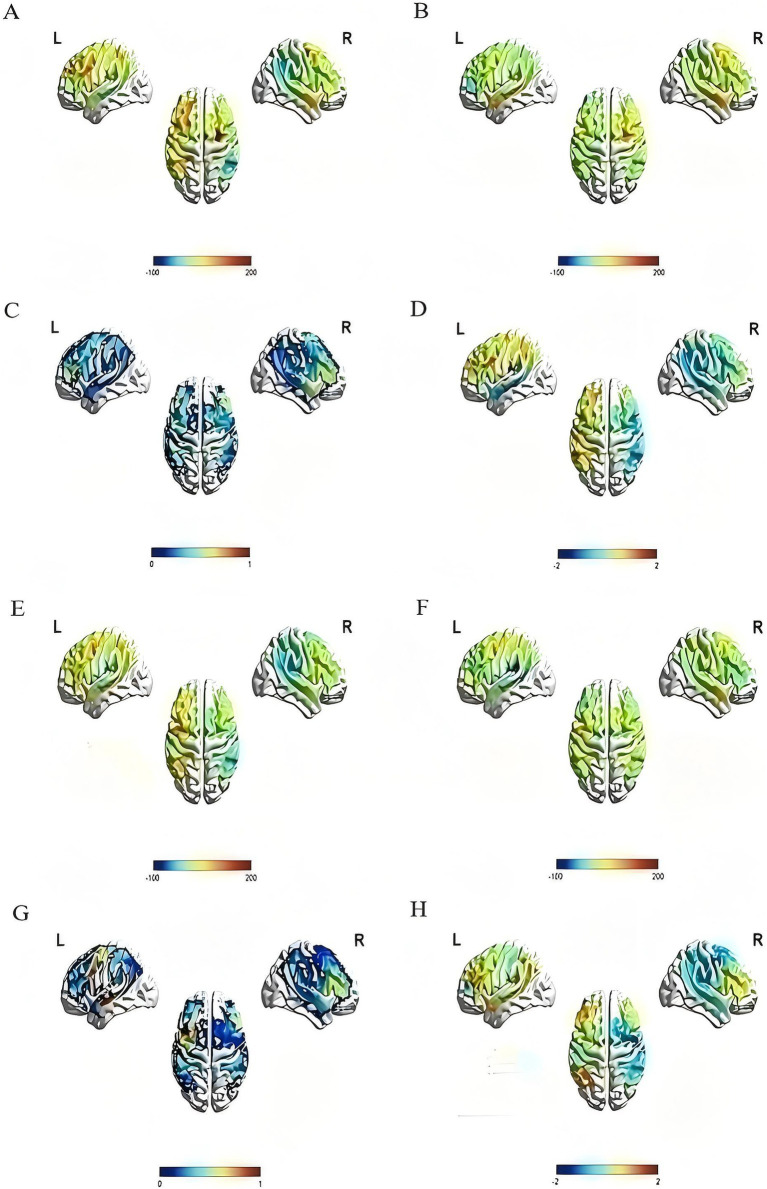
Activation of the rTMS group and the sham group before and after treatment. The color bar at the bottom of each subfigure represents the statistical size of beta value, *p* value or *T* value. The larger the beta value is, the stronger the activation relative to the baseline, and *p* < 0.05 is considered statistically significant. (A) The pre-treatment beta value of the rTMS group; (B) the post-treatment beta value of the rTMS group, compared with (A), it can be seen that brain activation in multiple regions decreased after treatment; (C) the *p* value diagram of the rTMS group after treatment; (D) the *T* value diagram of the rTMS group after treatment; (E) the pre-treatment beta value of the sham group; (F) the post-treatment beta value of the sham group, compared with (E), it can be seen that brain activation in multiple regions decreased after treatment; (G) the *p* value diagram of the sham group after treatment; (H) the *T* value diagram of the sham group after treatment.

The changes in activation of brain areas in the image naming task were reflected by the change in the beta value of each channel and ROI before and after the intervention. After 4 weeks of treatment, the beta values of channels 22 and 31 in the rTMS group were noticeably lower than those before treatment (channel 22: *t* = 3.541, *p* = 0.008; Channel 31: *t* = 2.507, *p* = 0.037), the beta value of channel 12 in the sham group was also lower than before (*t* = 2.582, *p* = 0.036), and the difference was statistically significant. In the left dorsolateral prefrontal lobe, the beta value of the rTMS group after treatment was significantly lower than before (*t* = 2.710, *p* = 0.027). No statistically significant difference was found between the two groups (Figure 6 and Table 5).

**Table 5:** Relationships between the Brodmann area and channel coverage.

Channel	Brodmann area	Channel coverage
22 (L)	MTG	0.349
22 (L)	STG	0.651
31 (L)	PM and SMA	0.329
31 (L)	DLPFC	0.004
31 (L)	Pars triangularis Broca’s area	0.668
12 (R)	PM and SMA	1

MTG, middle temporal gyrus; STG, superior temporal gyrus; PM, premotor cortex; SMA, supplementary motor area; DLPFC, dorsolateral prefrontal cortex; L, left hemisphere; R, right hemisphere.

## 4 Discussion

We aimed to evaluate the effects of low-frequency rTMS combined with speech and language therapy on Broca’s aphasia in subacute stroke inpatients. We aimed to determine whether the rTMS treatment led to a better language and cognitive function than the sham group at different time points after 4 weeks of intervention, and explore relevant mechanisms by examining the changes in activation degree of specific language brain regions during the picture-naming task with fNIRS. The overall language and cognitive function of the rTMS group presented a better recovery trend at all time points compared to the sham group. At 3 months post-treatment, the degree of improvement in language and cognitive function was greater than that at 4 weeks post-treatment, especially in naming performance.

Additionally, the picture-naming task was applied to investigate the activation degree of several brain regions (defined as ROIs) with fNIRS. After treatment, we found weakened brain activation in the left superior temporal gyrus (STG), middle temporal gyrus (MTG), pars triangularis Broca’s area, premotor cortex (PM), supplementary motor area (SMA), and dorsolateral prefrontal lobe (DLPFC) during naming. A previous study (21) on naming tasks found decreased activation in these brain regions (except DLPFC) in stroke patients with global aphasia in the chronic phase (instead of the subacute phase as in the current study). Another study (19) detected weakened DLPFC activation and suggested that the left prefrontal cortex of the aphasics utilizes more oxygen than the nonaphasics during a naming task. However, neither study (19, 21) compared pre- and post-treatment results.

Healthy people only need a low level of activation in the left middle temporal gyrus, left STG, and left pars triangularis Broca’s area during the picture-naming task (33). However, post-stroke aphasia patients need a higher level of brain activation during naming tasks (50–52). Therefore, our finding, i.e., weakened brain activation after rTMS treatment, indicates that for patients with post-stroke Broca’s aphasia after rTMS intervention, the magnitude of activation in these brain regions tends to approach the level of brain activation in healthy individuals. On the contrary, those in the sham group, who did not have rTMS and only received speech and language therapy, did not show such changes in these brain regions, and the degree of improvement in language and cognitive function of these individuals was also lower than that of the rTMS group.

In the present study, those strongly activated brain areas before treatment possibly highlighted ipsilateral compensation (53). This was required to exert certain language functions (54), possibly due to slowed information processing speed in the brain, which affected the coordination and integration between brain areas before naming. This results in a longer naming latency (55, 56).

rTMS can induce action potentials in neuronal axons (47) and modulate excitability of the targeted cortical regions as well as remote areas, thereby promoting brain function (14, 57). In the last decade, rTMS has been widely applied in post-stroke aphasia patients (58, 59). At present, the recovery mechanism of post-stroke aphasia may be explained by different theoretical models, such as interhemispheric inhibition and vicariation. The interhemispheric inhibition model suggests that mutual inhibition exists between brain hemispheres, and two hemispheres remain in a balanced state normally, whereas imbalance after stroke occurs, which leads to overexcitability of the right (unaffected) hemisphere (57). Therefore, downregulating the excitability of the unaffected hemisphere is necessary. Thus, if the interhemispheric inhibition model (57) is used for neuroregulation, the unaffected hemisphere should be inhibited (60). The second model, vicariation, suggests that the unaffected hemisphere takes over the work of the damaged regions and contributes to the improvement of overall language function after stroke (61). These two models of reorganization lead to opposite approaches, until the bimodal balance-recovery model was introduced (62), which combines these two models based on the structural reserve to apply optimal neuromodulatory strategies.

rTMS is non-invasive, effective, and safe if properly applied (16, 63, 64). Effects of rTMS on language and cognitive function have been confirmed by a growing body of research (65, 66). Low-frequency rTMS is used to downregulate/inhibit the neuronal responsiveness of cortical activity (7, 14, 66, 67). As suggested by the theory of interhemispheric inhibition, low-frequency rTMS of the right inferior frontal gyrus is beneficial to the recovery of non-fluent aphasia at the chronic or subacute stage, especially if combined with speech and language therapy (1, 44). For patients with aphasia, low-frequency rTMS produces immediate as well as long-term benefits, whereas high-frequency rTMS only presents long-term benefits (1, 26). Thus, in this study, we applied low-frequency rTMS (1 Hz) to the right pars triangularis Broca’s area. In post-stroke aphasia, low-frequency rTMS has been used to limit the recruitment of cerebral language networks of the unaffected (right) hemisphere to favor intrahemispheric compensation, which is related to better recovery of language functions (7, 14, 66, 67). The utilization of low-frequency rTMS was expected to reduce inhibition exerted by the right (unaffected) hemisphere over the left hemisphere, thereby mitigating the imbalance of mutual inhibition. Our results also show that the modulated brain activity evoked by rTMS accompanies better functional recovery at the behavioral level after 4 weeks of treatment.

One interesting finding in our study was the delayed positive treatment effect, which was observed at the time point 3 months after the intervention. Improvements in language ability were not observed (68, 69) until 2 months after the 10-session rTMS treatment in patients with chronic non-fluent aphasia. Another study on subacute stroke stages also reported improvement detected only 30 days after the intervention (15). In our study, the 3-month delayed effect might result from the dynamics underlying treatment-related neuroplasticity. Low-frequency rTMS may initially trigger a small potential treatment effect (15), whereas with subsequently activated pathways for recovery (38), abscopal effects, and more generated brain-derived influencing factor (BDNF) (65, 70) in subacute stages, the delayed effect occurs (71).

On the other hand, although multiple studies (38, 41, 42) have shown intensive speech and language therapy alone is effective in the chronic and subacute phase of aphasia (72), evidence shows the need for high doses and intensity (39). A previous meta-analysis (42) reported that the greatest clinical overall language and functional communication gains are associated with 2 to 4 and 9+ hours of speech and language therapy per week. However, discrepancies remain regarding clinical research and current routine rehabilitation services for optimal speech and language therapy regimens (73). Furthermore, clinical service reports describe an average of 60–90 min of speech and language therapy weekly for patients during early subacute stages of aphasia and 4–16 h as a total dosage (42). In our present study, according to the recommendations of practice guidelines and consensus on clinical management of post-stroke aphasia (40), combined with the clinical feasibility of patient treatment and following the rTMS procedure to ensure speech treatment within the period of optimal rTMS after-effect (about 45 min) (15), the speech and language therapy protocol was designed for 20 sessions, 30 min/session, 5 times a week (41). In clinical aphasia strategies, low-frequency rTMS is preferred when combined with speech and language therapy (41), and our results prove that such speech and language therapy protocols can result in functional improvement when combined with low-frequency rTMS.

We suggest that the improvement in naming ability after 4 weeks of treatment does not depend on local effects of stimulating a brain region, but is more likely to reflect the optimization of interregional connectivity. The functional connection between brain regions provides pathways by which stimulation of the right brain region causes decreased activation of multiple brain regions on the left side. A similar opinion was suggested in previous studies (9, 74): increased interhemispheric connectivity has a positive effect on naming tasks, and the brain tends to generate the least amount of activation to perform tasks efficiently and accurately. We, therefore, argue that overall recovery mainly stems from the intrahemispheric and interhemispheric functional recombination (53, 54, 75). Further studies are needed to elucidate the neurophysiological mechanisms underlying the degree of brain activation, functional connectivity between brain regions, and clinical behavioral manifestations during different language function tasks.

This study has limitations. The absence of follow-up beyond 3 months limits the ability to assess long-lasting effects. Additionally, the lack of neuro-navigation during transcranial magnetic stimulation treatment may prevent accurate localization.

## 5 Conclusion

We utilized low-frequency rTMS combined with speech and language therapy in subacute stroke patients with Broca’s aphasia. The clinical language function in the patients was improved after 4 weeks of treatment, and there was a long-term effect (3 months post-treatment), especially in naming gains. For the first time, we found that rTMS could improve language ability and cognitive function in patients with Broca’s aphasia in the subacute phase.

Moreover, by analyzing cortical activation during a picture-naming task with fNIRS, we found that rTMS could downgrade the activation level in the left MTG, STG, PM and SMA, DLPFC, and pars triangularis Broca’s area, whereas the sham-rTMs group only showed downgraded activation levels in the right PM and SMA. This demonstrates the unique mechanism of rTMS. We suggest that the improvement of language and cognitive functions in patients with Broca’s aphasia is related to intrahemispheric and interhemispheric functional reorganization. Additionally, the rTMS-induced down-regulated activation levels in the left DLPFC in subacute stroke patients with Broca’s aphasia has not been reported in other studies.

## Data Availability

The original contributions presented in the study are included in the article/supplementary material, further inquiries can be directed to the corresponding author.
